# ASSOCIATION OF LIVING ALONE AND SOCIAL ISOLATION WITH ALL-CAUSE AND CARDIOVASCULAR MORTALITY: THE OTASSHA STUDY

**DOI:** 10.1093/geroni/igae098.3226

**Published:** 2024-12-31

**Authors:** Keigo Imamura, Hisashi Kawai, Manami Ejiri, Hiroyuki Sasai, Hirohiko Hirano, Yoshinori Fujiwara, Kazushige Ihara, Shuichi Obuchi

**Affiliations:** Tokyo Metropolitan Institute for Geriatrics and Gerontology, Tokyo, Japan; Tokyo Metropolitan Institute for Geriatrics and Gerontology, Tokyo, Japan; Tokyo Metropolitan Institute for Geriatrics and Gerontology, Tokyo, Japan; Tokyo Metropolitan Institute for Geriatrics and Gerontology, Tokyo, Japan; Tokyo Metropolitan Institute for Geriatrics and Gerontology, Tokyo, Japan; Tokyo Metropolitan Institute for Geriatrics and Gerontology, Tokyo, Japan; Hirosaki University, Hirosaki, Aomori, Japan; Tokyo Metropolitan Institute for Geriatrics and Gerontology, Tokyo, Japan

## Abstract

Living alone is considered a risk factor for all-cause and cardiovascular mortality, but this association may be confounded by social isolation status. This study examined how living alone while also being socially isolated affects older adults’ risk of all-cause and cardiovascular mortality. A total of 4,075 community-dwelling older adults (mean age: 72 years, male: 46%) who had participated in the 2012 or 2013 mail survey of the health checkup of the Otassha Study were included; the study period was October 2012 to November 2020. Social isolation was assessed by frequency of interaction with others, and less than once a week was defined as social isolation. Participants were classified into four groups: no social isolation – not living alone, no social isolation – living alone, social isolation – not living alone, and social isolation – living alone. A Cox proportional hazards model was used to analyze the association of combined living alone and social isolation with all-cause and cardiovascular mortality. A total of 203 (5.0%) of the participants were found to be living alone while being socially isolated. Social isolation was significantly associated with all-cause and cardiovascular mortality, regardless of whether the participants lived alone or not (p< 0.05). On the other hand, there was no association with mortality among those who lived alone but were not socially isolated. These results indicate that living alone is not necessarily a risk factor for all-cause or cardiovascular mortality. In conclusion, engaging in regular social interactions could help older adults reduce mortality.

